# Optic nerve head blood flow regulation during changes in arterial blood pressure in patients with primary open‐angle glaucoma

**DOI:** 10.1111/aos.13850

**Published:** 2018-09-14

**Authors:** Ahmed M. Bata, Klemens Fondi, Katarzyna J. Witkowska, René M. Werkmeister, Anton Hommer, Clemens Vass, Hemma Resch, Doreen Schmidl, Alina Popa‐Cherecheanu, Jacqueline Chua, Gerhard Garhöfer, Leopold Schmetterer

**Affiliations:** ^1^ Department of Clinical Pharmacology Medical University of Vienna Vienna Austria; ^2^ Center for Medical Physics and Biomedical Engineering Medical University of Vienna Vienna Austria; ^3^ Department of Ophthalmology Sanatorium Hera Vienna Austria; ^4^ Department of Ophthalmology Medical University of Vienna Vienna Austria; ^5^ Department of Ophthalmology Emergency University Hospital Bucharest Romania; ^6^ Carol Davila University of Medicine and Pharmacy Bucharest Romania; ^7^ Singapore Eye Research Institute Singapore Singapore; ^8^ Lee Kong School of Medicine Nanyang Technological University Singapore Singapore; ^9^ Ophthalmology and Visual Sciences Academic Clinical Program Duke‐NUS Medical School Singapore Singapore

**Keywords:** glaucoma, laser doppler flowmetery, laser Doppler flowmetry, optic nerve head autoregulation, optic nerve head blood flow

## Abstract

**Purpose:**

Abnormal autoregulation of optic nerve head blood flow (ONHBF) has been postulated to play an important role in primary open‐angle glaucoma (POAG). We used laser Doppler flowmetry (LDF) to estimate quantitatively the ONHBF and compared ONHBF autoregulation between glaucoma patients and healthy controls during isometric exercise.

**Methods:**

Forty patients with POAG and 40 healthy age‐ and sex‐matched subjects underwent three periods of isometric exercise, each consisting of 2 min of handgripping. Optic nerve head blood flow (ONHBF) was measured continuously using LDF. Systemic blood pressure, intraocular pressure and ocular perfusion pressure were assessed in all participants.

**Results:**

Isometric exercise was associated with an increase in ocular perfusion pressure during all handgripping periods in both groups (p < 0.001). However, there was no change in ONHBF in either group. Three of the glaucoma patients and two of the healthy subjects showed a consistent 10% decrease in blood flow during isometric exercise, in spite of an increase in their blood pressure. This difference between groups was not significant (p = 0.61). Four other glaucoma subjects showed a consistent increase in blood flow of more than 10% during isometric exercise, whereas this was not seen in healthy subjects (p = 0.035).

**Conclusion:**

This study suggests that abnormal ONHBF autoregulation is more often seen in patients with POAG than healthy control subjects. The relationship to the glaucoma disease process is currently unknown and requires further investigation.

## Introduction

Abnormal autoregulation of the optic nerve head blood flow (ONHBF) has been implicated in the pathophysiology of primary open‐angle glaucoma (POAG) (Flammer & Mozaffarieh 2008; Harris et al. 2008; Cherecheanu et al. 2013). However, direct experimental evidence of this autoregulatory behaviour in the human optic nerve head (ONH) is sparse (Schmidl et al. 2011). Only few studies looked into the association between ocular perfusion pressure (OPP) and blood flow parameters at the posterior pole of the eye and found abnormal patterns in patients with POAG (Fuchsjager‐Mayrl et al. 2004; Portmann et al. 2011).

Measuring ONH blood flow (ONHBF) autoregulation is challenging. Firstly, it is technically difficult because of the complex angio‐architecture of the optic nerve head region (Mackenzie & Cioffi 2008). Until now, two approaches have been attempted to quantify blood flow, namely laser Doppler flowmetry (LDF) (Riva et al. 2010) and laser speckle flowgraphy (Sugiyama et al. 2010). The second challenge relates to the stimuli used to modify OPP, because many interventions are too invasive to be used in elderly patients with eye disease. Both isometric exercise (Movaffaghy et al. 1998; Schmidl et al. 2012, 2013a; Boltz et al. 2013a,b,c) and postural change (Shiga et al. 2013) have been used to increase OPP. Experimental increase in intraocular pressure (IOP) (Pillunat et al. 1997; Riva et al. 1997; Schmidl et al. 2012, 2013b; Boltz et al. 2013c; Hashimoto et al. 2017a,b) and thigh‐cuff release (Ikemura et al. 2015) has been used to decrease OPP in humans.

Majority of studies have only recruited healthy subjects because these interventions are cumbersome for elderly patients with ocular diseases to perform. We have now developed a protocol using handgripping, a form of isometric exercise that can be safely achieved by the elderly. In this study, we investigated whether patients with POAG have abnormal autoregulation of ONHBF during isometric exercise compared to healthy age‐ and sex‐matched control subjects.

## Materials and Methods

This study was approved by the Ethics Committee of the Medical University of Vienna and was performed in accordance with the Declaration of Helsinki and the Good Clinical Practice (GCP) guidelines. Forty patients with POAG and 40 healthy age‐ and sex‐matched control subjects were included in this study. All participants signed written informed consent after the nature of the study had been explained in detail. Each subject passed a screening examination that included medical history and physical examination. Subjects were excluded if they were pregnant, were smokers, had uncontrolled hypertension or if any abnormality was found as part of the pretreatment screening unless the investigators considered the abnormality to be clinically irrelevant.

Patients with POAG were recruited from the outpatient clinic of the glaucoma service at the Department of the Ophthalmology, Medical University of Vienna Austria, and the private praxis of Dr. Anton Hommer. The healthy control subjects were recruited from the database of the Department of Clinical Pharmacology, Medical University of Vienna, Austria. All participants underwent a standardized ophthalmic examination, including slit lamp biomicroscopy, indirect funduscopy, gonioscopy and applanation tonometry. In subjects with childbearing potential a pregnancy test was performed. Diagnosis of manifest POAG was defined as pathological optic disc appearance, glaucoma hemifield test outside normal limits and pathological thinning of retinal nerve fibre layer as shown in optical coherence tomography (OCT‐RNFL). Glaucoma patients were excluded if they met these criteria: exfoliation glaucoma, pigmentary glaucoma, history of acute angle closure, mean deviation (MD) of visual field testing (Humphery VFA, 30‐2 with near correction, SITA pac) worse than −10, intraocular surgery or laser trabeculoplasty within the last 6 months, ocular inflammation or infection within the last 3 months and pregnancy. Further exclusion criteria were ocular disease other than glaucoma potentially interfering with the purposes of the study as judged by the investigators. Healthy control subjects were age‐ and sex‐matched and had to have normal ophthalmic findings, that is no evidence of increased IOP in previous ocular history as well as no structural and/or functional signs of glaucomatous damage and IOP <20 mmHg on at least three measurements.

Sample size calculation was performed according to previous reproducibility data in healthy subjects (Boltz et al. 2013c). The standard variation for ONHBF is approximately 15%. Using an alpha error of 5% and a beta error of 80% a sample size of 30 in each group is required to detect differences between groups in the order of 10%. Differences smaller than this were thus considered irrelevant. In order to account for drop‐outs, we included a total of 40 subjects per group.

### Experimental design

The protocol followed the schedule we have previously used in healthy subjects (Boltz et al. 2013c). Dilatation of the pupil of the right eye was obtained with topical tropicamide (Mydriaticum Agepha‐Augentropfen, Vienna, Austria). A resting period of at least 20 min in a sitting position was scheduled to ensure sufficient mydriasis and stabilization of blood pressure. Intraocular pressure (IOP), blood pressure and pulse rate were measured at the beginning and at the end of the session. Optic nerve head blood flow (ONHBF) was analysed continuously with LDF throughout the entire session, that is during resting periods and isometric exercise. The experiments consisted of the following sequence: rest (20 min) – handgrip (2 min) – rest (2 min) – handgrip (2 min) – rest (2 min) – handgrip (2 min) – rest. Blood pressure was also measured every minute throughout the study period. The main outcome parameter of the study was the number of subjects that showed an increase or a decrease in more than 10% during handgripping periods. This was chosen based on our previous data in healthy subjects (Boltz et al. 2013c).

### Measurements

#### Blood pressure and pulse rate

Systolic blood pressure (SBP), diastolic blood pressure (DBP) and mean arterial blood pressure (MAP) were measured on the upper arm by an automated oscillometer. Pulse rate (PR) was recorded from a finger pulse‐oxymetric device (HP‐CMS patient monitor, Hewlett Packard, Palo Alto, CA, USA).

#### Laser Doppler flowmetry (LDF)

Measurements were performed using a laser Doppler flowmeter for ONHBF (Oculix 4000, Oculix, Arbaz, Switzerland). Briefly, the Doppler shift power spectrum is used to measure blood flow, blood velocity and blood volume based on a theory of light scattering in tissue. Velocity is calculated as the mean velocity of the red blood cells moving in the sampled tissue proportional to the mean Doppler frequency shift. Volume is the number of moving red blood cells in the sampled tissue proportional to the amount of Doppler shifted light. Blood flow is calculated as the product of velocity and volume. Only data with a direct current (DC) value of ±15% to the baseline value were included for analysis. Subjects were excluded from analysis if they did not meet this reproducibility criterion during each of the 2‐min period. The laser beam was directed towards the temporal neurovascular rim, and any visible vessels within the scattering volume were avoided. Data were corrected for differences in DC as published previously (Pemp et al. 2009).

#### Intraocular pressure and ocular perfusion pressure

Intraocular pressure (IOP) was measured using Goldmann applanation tonometer. Ocular perfusion pressure in the sitting position was calculated as 2/3* MAP – IOP (Robinson et al. 1986).

#### Isometric exercise

Subjects were instructed to rest their forearm on a table with the elbow at an angle of 90° during muscular contraction. The isometric handgrip was performed for 120 seconds at 75% of previously determined individual maximal voluntary contraction using a handgrip dynamometer.

### Data analysis

An independent *t*‐test was performed for continuous variables, and chi‐square test was used for categorical variables. A repeated‐measures anova model was used to analyse data at various timepoints. A multivariate linear regression models with generalized estimating equations investigated the effect of age, gender, IOP, systemic blood pressure, pulse rate, presence of systemic hypertension and presence of hypercholesterolemia, and type of topical antiglaucoma medication. We also analysed individual's ONHBF traces during isometric exercise or the recovery phase as seen in our previous experiments (Boltz et al. 2013c) We categorized individuals as having abnormal ONHBF autoregulation as those having an increase or a decrease in more than 10% during handgripping periods. This outcome was chosen as the main outcome parameter of the present study. To determine a change, percentage change was calculated from baseline values. In addition, we investigated whether patients with abnormal responses differed from those with normal response in terms of age, gender, IOP, visual field defect, retinal nerve fibre layer thickness, systemic blood pressure, pulse rate, presence of systemic hypertension and presence of hypercholesterolemia, and type of topical antiglaucoma medication. A p‐value <0.05 was considered the level of significance. Statistical analysis was carried out using CSS statistica for Windows^®^ (Statsoft Inc., Version 6.0, Tulsa, CA, USA).

## Results

Baseline characteristics of the participants are presented in Table 1. The most frequent systemic diseases in both groups were systemic hypertension and hypercholesterolemia. Whereas systemic hypertension was more frequent in patients with glaucoma than in healthy controls (POAG: *n* = 18, controls: *n* = 7; p = 0.09), there was no difference in the frequency of hypercholesterolemia (POAG: *n* = 8, controls: *n* = 6; p = 0.14). There was no difference in age or gender distribution between the two groups. Systolic blood pressure was slightly higher in POAG patients than in healthy control subjects. Table 2 shows the type of topical anti‐glaucoma treatment used in the POAG group. Among the participants, 32 POAG and 33 healthy control subjects met the LDF reproducibility criterion. Older participants tended not to achieve good reproducible LDF readings (p = 0.02).

**Table 1:** Demographic and baseline characteristics of the subjects participating in the study. Data are presented as means ± SD (*n* = 40 per group)

	Primary open‐angle glaucoma patients	Healthy control subjects	p Value
Age (years)	58.9 ± 12.2	58.8 ± 12.5	0.97
Sex (M/F)	14/26	14/26	1.0
Systolic blood pressure (mmHg)	139.7 ± 19.6	130.8 ± 14.8	0.03
Diastolic blood pressure (mmHg)	81.2 ± 10.7	78.1 ± 8.7	0.16
Mean arterial pressure (mmHg)	107.6 ± 13.4	102.1 ± 10.7	0.05
Pulse rate (beats per minute)	65.4 ± 10.9	64.6 ± 8.5	0.72
Intraocular pressure (mmHg)	13.1 ± 2.7	12.7 ± 2.9	0.53
Ocular perfusion pressure (mmHg)	57.2 ± 7.1	53.2 ± 6.7	0.01
Optic nerve head blood flow (arbitrary units)	19.7 ± 6.8	24.5 ± 6.3	0.002
Retinal nerve fibre layer thickness (*μ*m)	73.3 ± 13.6	97.0 ± 11.0	<0.001
Mean defect (dΒ)	−3.7 ± 3.0	−0.4 ± 1.0	<0.001

**Table 2:** Treatment characteristics of patients with primary open angle glaucoma (*n* = 40)

	Number of patients (*n*)
History of trabeculectomy	8
History of selective laser trabeculoplasty	1
No topical therapy	4
Topical therapy with beta receptor blockers	24
Topical therapy with prostaglandin analogues	32
Topical therapy with carboanhydrase inhibitors	16
Topical therapy with alpha receptor agonists	1
Topical therapy with one drug class	14
Topical therapy with two drug classes	6
Topical therapy with three drug classes	16

**Figure 1:**
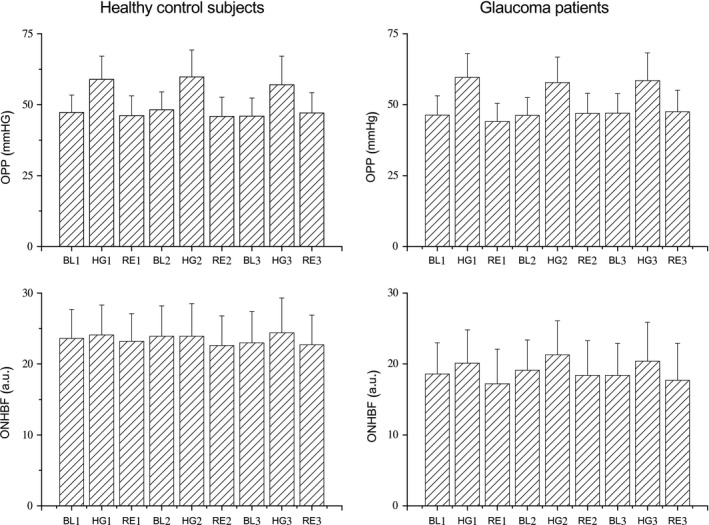
Effect of handgripping on ocular perfusion pressure (OPP) and optic nerve head blood flow (ONHBF). Three periods of isometric exercise were scheduled for each subject (*n* = 39). Data are presented before during and after the 1st handgripping experiment (BL1, HG1, RE1), before during and after the 2nd handgripping experiment (BL2, HG2, RE2) and before during and after the 3rd handgripping experiment (BL3, HG3, RE3). In the boxplots, the means and standard deviations are shown.

The effect of isometric exercise on MAP and PR is shown in Table 3. All participants showed a significant increase in MAP and PR during isometric exercise (p < 0.001 each, anova). There was no significant difference between POAG patients and healthy control subjects (MAP: p = 0.84; PR: p = 0.70). The effect on OPP and ONHBF is depicted in Fig. 1. Similarly, the two groups did not show significant difference in their OPP (p = 0.55) and ONHBF (p = 0.11). Baseline ONHBF was, however, higher in healthy control subjects (22.7 ± 4.2 a.u.) than in POAG patients (18.3 ± 4.6 a.u., p < 0.001). Intraocular pressure did not change in the present experiments (baseline: 13.9 ± 1.4 mmHg; end of study: 13.5 ± 1.2 mmHg, p = 0.68). In the multivariate linear regression model, none of the tested factors was associated with the blood flow response to isometric exercise (p > 0.1 for all variables, data not shown).

**Table 3:** Effects of isometric exercise (handgripping, 3 periods) on mean arterial pressure (MAP), pulse rate (PR) and ocular perfusion pressure (OPP) in all participating subjects (primary open‐angle glaucoma (POAG) *n* = 32, healthy control subjects *n* = 33). Data are presented as means ± SD

	Before 1st period	During 1st period	After 1st period	Before 2nd period	During 2nd period	After 2nd period	Before 3rd period	During 3rd period	After 3rd period
POAG patients									
MAP (mmHg)	91.7 ± 8.4	105.6 ± 11.1	90.1 ± 8.8	92.5 ± 8.0	104.4 ± 12.3	92.1 ± 8.5	92.1 ± 8.4	104.1 ± 11.7	93.1 ± 9.0
PR (beats per minute)	71.5 ± 10.4	82.2 ± 13.6	71.4 ± 10.7	72.3 ± 11.2	81.9 ± 13.6	71.4 ± 10.7	71.5 ± 10.4	82.2 ± 13.6	71.4 ± 10.7
Healthy controls									
MAP (mmHg)	92.4 ± 8.0	103.6 ± 10.6	91.4 ± 8.4	93.4 ± 8.8	104.4 ± 12.1	91.1 ± 8.2	91.6 ± 8.0	103.1 ± 11.5	93.6 ± 9.3
PR (beats per minute)	71.0 ± 12.0	83.2 ± 12.2	72.0 ± 12.2	71.6 ± 11.6	83.5 ± 12.8	71.4 ± 12.7	70.8 ± 13.3	82.8 ± 13.3	72.5 ± 12.1

Individuals exhibiting changes in ONHBF of more than 10% were observed (Table 4). Four glaucoma patients showed a consistent 10% increase in ONHBF during isometric exercise, whereas this was not seen in healthy subjects (p < 0.035). A decrease in ONHBF was consistently seen in two healthy subjects and three glaucoma patients during isometric exercise, but this difference was statistically not significantly (p = 0.62). In the recovery period, none showed an increase in ONHBF. In contrast, a consistent decrease in more than 10% was observed in three POAG patients and two healthy control subjects during the recovery period. This difference between groups was not significant (p = 0.62). Glaucoma patients showing a consistent 10% increase in ONHBF during isometric exercise did not differ in terms of age, gender, IOP, visual field defect, retinal nerve fibre layer thickness, systemic blood pressure, pulse rate, presence of systemic hypertension and presence of hypercholesterolemia and type of topical antiglaucoma medication (data not shown).

**Table 4:** Frequency of abnormal blood flow changes of more than 10% in all participating subjects (primary open‐angle glaucoma (POAG) *n* = 32, healthy control subjects *n* = 33)

Change in blood flow	Number of subjects
*During handgripping*	
POAG	
Decrease in more than 10% during 1, 2, or all 3 periods	1/1/3
Increase in more than 10% during 1, 2, or all 3 periods	1/0/4
Healthy controls	
Decrease in more than 10% during 1, 2, or all 3 periods	1/0/2
Increase in more than 10% during 1, 2, or all 3 periods	0/1/0
*During recovery*	
POAG	
Decrease in more than 10% during 1, 2, or all 3 periods	2/1/3
Increase in more than 10% during 1, 2, or all 3 periods	1/0/0
Healthy controls	
Decrease in more than 10% during 1, 2, or all 3 periods	3/1/2
Increase in more than 10% during 1, 2, or all 3 periods	2/1/0

## Discussion

In the present study, we observed that a proportion of POAG patients showed an abnormal ONHBF autoregulation when compared to healthy control subjects. Changes in OPP during handgripping periods and ONHBF autoregulatory range were similar in the whole‐population sample (Fig. 1). However, a subgroup of POAG subjects showed abnormal blood flow responses during the isometric exercise. As such the present study may be a first step in detecting early signs of ONHBF autoregulatory disturbances may be of value in some POAG individuals.

A number of previous studies indicated abnormal ocular blood flow autoregulation in glaucoma using different technologies for the assessment of ocular perfusion. Many of these studies used measurement of retrobulbar flow velocities with colour Doppler imaging (CDI) to gain insight into ocular hemodynamics (Evans et al. 1999; Gherghel et al. 2000; Garhofer et al. 2010). The problem is, however, that with this technique blood velocities in the retrobulbar circulation are measured and their relation to ONHBF is unclear (Stalmans et al. 2009; Dimitrova & Kato 2010). Moreover, resistance index, a parameter calculated from CDI measurements does not seem to adequately reflect changes in vascular resistance (Polska et al. 2001). Investigations on retinal blood flow autoregulation have been reported using laser Doppler velocimetry (Feke & Pasquale 2008), and the blue field entoptic system (Grunwald et al. 1984), fluorescein angiography (Plange et al. 2008) and retinal vessel analysis (Nagel et al. 2001) and have consistently identified abnormal autoregulation in glaucoma. Single‐point LDF as used in the present study was previously shown to identify abnormalities in choroidal blood flow autoregulation during isometric exercise (Portmann et al. 2011).

The number of studies that focused specifically on ONHBF is small. Variations of ONHBF during diurnal variations in OPP were studied using scanning LDF (Sehi et al. 2005). The regions of greatest diurnal change in rim topography showed significant diurnal change in blood flow in patients with POAG but not in normal controls. Using the same technique, an abnormal association between ONHBF and OPP was observed in glaucoma patients (Fuchsjager‐Mayrl et al. 2004), which was normalized after initiating IOP‐lowering therapy (Fuchsjager‐Mayrl et al. 2010). Interestingly this normalization was seen after pressure reduction with both timolol and dorzolamide, which induced different vasomotor effects at the posterior pole of the eye. This indicates that increased IOP itself can induce changes in autoregulation, a hypothesis that has gained support by a number of studies (Schmidl et al. 2011; Boltz et al. 2013b). In the present study, this is unlikely to explain the results, because IOP was not significantly different between groups.

A technique related to LDF is laser speckle flowgraphy (Sugiyama et al. 2010), although some significant differences exist between the two methods (Witkowska et al. 2017) Reduced ONHBF (Yaoeda et al. 2003; Kobayashi et al. 2014; Shiga et al. 2016) as well as reduced blood flow response to hyperoxia (Kiyota et al. 2017) has been reported in glaucoma patients. To the best of our knowledge, the technique has not yet been used to characterize autoregulation in glaucoma. In a study in non‐human primates with experimentally increased IOP changes in ONH autoregulation were, however, reported (Wang et al. 2014, 2015).

The present study has some strength and weaknesses: we used repeated continuous stimuli which hopefully better identify individuals with abnormal blood flow response rather than solely relying on group averages. We had a relatively small number of patients in the present study. This, in part, is related to the difficulty of operating the LDF. In addition, we chose a margin of 10% change in blood flow as having an autoregulatory response. This is based on our previous data (Boltz et al. 2013c), but we do currently not know whether this is a clinically meaningful margin. Whereas we did 3 repeated OPP challenges and were able to identify consistent changes in different subjects we do not know the day‐to‐day reproducibility or the long‐term reproducibility of our results. This needs to be studied in future longitudinal trials also testing the hypothesis that abnormal autoregulatory patterns are associated with glaucoma progression.

Also, we could only reliably obtain blood flow information from approximately 80% of subjects which indicates that the current technique is not ideal for clinical setting (Luksch et al. 2009). Future studies can consider using the commercialized optical coherence tomography angiography (OCTA), which is relatively easy to perform and less time consuming than LDF, together with the handgripping protocol to study blood flow (Kashani et al. 2017). However, algorithms will need to be developed to extract clinically valid perfusion information from OCTA (Leitgeb et al. 2014; Chen & Wang 2017). Majority of the POAG patients received topical anti‐glaucoma drugs. This may influence the results of the present study, but the multivariate analysis did not identify any differences between the different drugs. The diversity of treatment regimen was, however, large, and studies with larger sample size are required to study this topic in more details. As mentioned above, the strict IOP control does, however, have the advantage that the two study groups were matched with regard to IOP. Finally, the increase in blood pressure during handgripping is small and within the autoregulatory range in the majority of subjects in this study. As such we also did not observe a significant difference between the two groups. On the other hand, interventions that induce large blood pressure changes may not be well‐tolerated by older subjects.

In conclusion, the present study provides direct evidence for altered ONHBF regulation in some POAG patients. Using repeated stimuli with isometric exercise, we were able to identify subjects with abnormal ONHBF regulation patterns and these subjects were more frequently identified in the glaucomatous group than in the healthy control group. There is a need to further refine this technique to improve its feasibility for the clinic, thereby allowing future studies to investigate the autoregulatory abnormalities in the disease process.
